# Crystal Facet Engineering of 2D SnSe_2_ Photocatalysts for Efficient Degradation of Malachite Green Organic Dyes

**DOI:** 10.3390/nano15110850

**Published:** 2025-06-02

**Authors:** Liying Wen, Fangfang Cheng, Xinyu Zhao, Lin Han, Dongye Zhao, Shifeng Wang

**Affiliations:** 1 Key Laboratory of Plateau Oxygen and Living Environment of Xizang Autonomous Region, College of Science, Xizang University, Lhasa 850000, China; wenliying@stu.utibet.edu.cn (L.W.); chengfangfang@stu.utibet.edu.cn (F.C.); zhaoxinyu@stu.utibet.edu.cn (X.Z.); hanlin@stu.utibet.edu.cn (L.H.); zdy@utibet.edu.cn (D.Z.); 2 School of Ecology, Xizang University, Lhasa 850000, China

**Keywords:** SnSe_2_, photocatalytic properties, malachite green, crystal facet engineering

## Abstract

Wastewater containing triphenylmethane dyes such as malachite green (MG), discharged by textile and food industries, poses significant carcinogenic risks and ecological hazards. Conventional physical adsorption methods fail to degrade these pollutants effectively. To address this challenge, we focused on two-dimensional SnSe_2_ semiconductor materials. While their narrow bandgap and unique structure confer exceptional optoelectronic properties, prior research has predominantly emphasized heterojunction systems. We synthesized SnSe_2_ with well-defined hexagonal plate-like structures via a one-step hydrothermal method by precisely controlling precursor ratios (Sn:Se = 1:2) and reaction temperatures (120–240 °C). Systematic investigations revealed that hydrothermal temperature modulates the van der Waals forces between crystal planes, enabling selective exposure of (001) and (011) facets, as confirmed by XRD, SEM, and XPS analyses, thereby influencing the exposure of specific crystal facets. Experiments demonstrated that pure SnSe_2_ synthesized at 150 °C achieved complete degradation of MG (40 mg/L) within 60 min under visible light irradiation, exhibiting a reaction rate constant (k) of 0.099 min⁻¹. By regulating the exposure ratio of the active (001)/(011) facets, we demonstrate that crystal facet engineering directly optimizes carrier separation efficiency, thereby substantially enhancing the catalytic performance of standalone SnSe_2_. This work proposes a novel strategy for designing noble-metal-free, high-efficiency standalone photocatalysts, providing crystal facet-dependent mechanistic insights for the targeted degradation of industrial dyes.

## 1. Introduction

Dye-contaminated wastewater discharged from textile and food industries, characterized by carcinogenicity, ecological contamination, and high chromaticity, has emerged as a critical challenge in global water pollution management [1]. MG, a triphenylmethane dye [2], exhibits acute toxicity and carcinogenicity, posing severe threats to ecosystems and organismal health [3,4,5]. Effective removal of these hazardous dyes from industrial wastewater is therefore imperative. While traditional physical adsorption methods merely transfer pollutants without degradation, photocatalytic technology recognized for its environmental compatibility, sustainability, and low energy consumption enables the complete mineralization of organic pollutants or their conversion into harmless substances, driving its widespread adoption. However, existing non-noble metal and single-component photocatalysts frequently exhibit suboptimal degradation efficiency [6].

Two-dimensional (2D) semiconductor materials demonstrate promising potential for environmental remediation and water purification applications [7,8]. Since the discovery of graphene in 2004, 2D materials have garnered significant attention due to their exceptional electronic properties, low density of dangling bonds, high specific surface area, remarkable mechanical flexibility, and optical transparency. Furthermore, their unique electrical, thermal, and magnetic properties originate from quantum confinement effects in two-dimensional spaces and suppressed interlayer electron scattering [9]. These attributes are critical for advancing technologies in optoelectronic devices, supercapacitors, sensors, electrocatalysts, photocatalysts, and solar cells [10,11]. Recently, 2D transition metal dichalcogenides (TMDCs) such as MoS_2_ and SnSe_2_ have emerged as prominent candidates owing to their narrow bandgap and high carrier mobility.

SnSe_2_, an *n*-type semiconductor classified in the IV–VIA group, exhibits a narrow bandgap of 1–2 eV. Its CdI_2_-type layered structure endows exceptional chemical stability, complemented by Earth abundance, low toxicity, and eco-friendliness, making it highly promising for thermoelectric [12,13,14] and optoelectronic applications [15,16,17,18]. However, research on SnSe_2_ as a photocatalyst has predominantly focused on heterojunction systems. For instance, Tan et al. [19] employed SnSe_2_ to enhance the photocatalytic activity of Ag_3_PO_4_ particles, while Li et al. [20] synthesized SnSe_2_/Se heterojunction films to evaluate their photocatalytic efficiency. Mu et al. [21] constructed a Se/SnSe_2_/TiO_2_ multi-heterojunction system for simultaneous photoelectrochemical degradation of rhodamine B (Rh B). Recent studies on MG degradation highlight the need to balance efficiency and stability in photocatalytic systems. While heterojunction-based catalysts dominate current research, their complexity and instability limit practical applications. Recent advances in photocatalytic degradation of MG highlight the critical challenge of balancing efficiency and stability between heterojunction-based and standalone catalysts [22,23,24,25].

To address the lack of systematic studies on the intrinsic catalytic properties of 2D SnSe_2_ without external co-catalysts, we employed a one-step hydrothermal synthesis strategy, modulated interplanar van der Waals interactions to regulate the exposure levels of specific crystal facets, and systematically investigated their photocatalytic activity toward MG degradation [26]. By correlating hydrothermal temperatures with exposed facet evolution, we identified optimal growth conditions enabling SnSe_2_ to achieve 100% MG degradation within 60 min. Significantly, this study provides the first experimental evidence that pristine SnSe_2_ can serve as a high-efficiency standalone photocatalyst through crystal facet engineering, offering a novel paradigm for designing noble metal-free photocatalytic systems.

## 2. Materials and Methods

### 2.1. Materials

The chemicals used include Tin (II) chloride dihydrate (SnCl_2_·2H_2_O), Selenium dioxide (SeO_2_), and hydrazine hydrate (N_2_H_4·_H_2_O), all purchased from Shanghai Aladdin Biochemical Technology Co., Ltd., Shanghai, China. Ultrapure water (UPW) used in the experiment was prepared using UPW ultrapure water system from Sichuan ULUPURE Company, Sichuan, China. All chemical reagents were used without further purification.

### 2.2. Preparation of SnSe_2_

**Figure 1:**
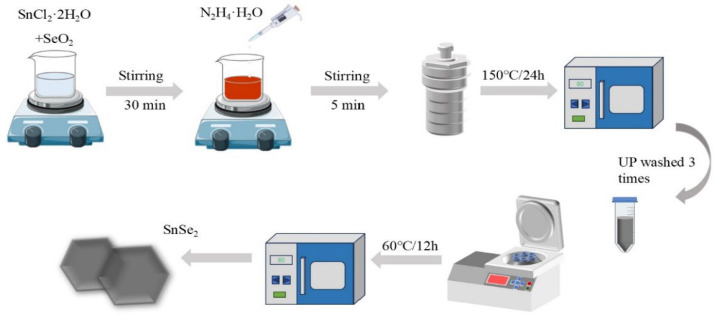
Flowchart for the preparation of SnSe_2_ materials.

SnSe_2_ was prepared by a hydrothermal method as follows [27,28]: firstly, 2 mmol of SnCl_2·_2H_2_O and 4 mmol of SeO_2_ were dissolved in 40 mL of deionized water, and the mixture was stirred at 400 rpm. After stirring for 30 min, 2 ml of N_2_H_4·_H_2_O was added, and the solution was stirred at 400 rpm for 5 min. The resulting solution was transferred into a hydrothermal autoclave (volume specification 100 mL) and heated at 120 °C, 150 °C, 180 °C, 210 °C and 240 °C for 24 h; the heating procedure was regulated by a programmed temperature control system. After cooling to room temperature (the cooling stage proceeded through natural convection without external thermal regulation), the samples were washed three times with UPW, followed by drying at 60 ℃ for 12 h. The resulting product was obtained as a black-colored powder. The experimental procedure is illustrated in Figure 1.

### 2.3. Characterizations

The phase composition and crystal structure of the samples were characterized using a Bruker D8 ADVANCE X-ray diffractometer (XRD, Billerica, MA, USA) with Cu Kα radiation (λ = 1.5406Å), under 40 kV and 40 mA conditions. The morphology and microstructure of the samples were probed using a HITACHI S-4800 scanning electron microscope (SEM, Hitachi, Ltd., Tokyo, Japan) and an FEI Talos F200X transmission electron microscope (TEM, Thermo Fisher Scientific, Waltham, MA, USA). The surface composition and electronic structure of the samples were determined using X-ray photoelectron spectroscopy (XPS, Thermo Fisher Scientific, Waltham, MA, USA), performed on a Thermo Escalab 250XI spectrometer with calibration of binding energies referenced to the C1s peak at 284.8 eV. Transient photocurrent response and impedance were measured using an electrochemical workstation (Shanghai Chenhua CHI700E, China, Universal Bipotentiostat, Shanghai, China), a saturated calomel reference electrode, platinum (Pt) counter electrode, and ITO glass working electrode. The sample (approximately 5 mg) was dispersed in a mixed solution containing 50 μL Nafion and 950 μL ethanol. For photocurrent measurements, a 300 W Xe lamp light source was used with a 400 s test duration (20 s intervals for on/off cycles). Electrochemical impedance analysis was performed with a frequency range of 0.1–100,000 Hz and an amplitude of 10 mV. The photoluminescence (PL – Edinburgh Instruments, EI, Edinburgh, UK) spectrum of the sample was measured using the FLS1000 fluorescence spectrometer, Edinburgh Instruments Ltd. Measurements of specific surface area (BET, WGB Sci & Tech Ltd. Beijing, China), pore size distribution, adsorption–desorption isotherms, and t-plot analysis were performed using a BK122W-01 analysis station.

A Xenon lamp (Solar-500, Beijing NBET Technology Co. Ltd., Beijing, China) was used as the light source in the experiment. The adsorption and photocatalytic activity of the SnSe_2_ material were evaluated using malachite green oxalate. A sample of 20 mg was dispersed in 100 mL of an MG oxalate solution with a concentration of 40 mg/L. The sample was sonicated in the dark for 30 min to achieve adsorption equilibrium (see Figure S2, Supplementary Materials). Under Xenon lamp irradiation, 6 mL of supernatant was taken every 10 min, and 3 mL of the solution was centrifuged and collected in a cuvette. Finally, the absorbance of the obtained liquid was measured at a wavelength of 617 nm using a UV–visible spectrophotometer (UV, INESAL5S, INASE Scientific Instrument CO., LTD., Shanghai, China). The work function (Φ) was measured using ultraviolet photoelectron spectroscopy (UPS, Thermo Fisher Scientific, Waltham, MA, USA). Calibrated UPS spectra were processed to determine the intercept value (X) via the tangential extrapolation method. As per the standard formula, the work function (Φ) was calculated as Φ = 21.22 eV − X.

The degradation efficiency of malachite green (MG) was calculated as [29]:

Degradation (%) = (1 − C/C_0_) × 100

where C and C₀ represent the real-time and initial dye concentrations, respectively.

The degradation kinetics under simulated sunlight followed a pseudo-first-order model [30]:

LnC_0_^′^/C = kt

where k is the rate constant (min⁻¹), C represent the real-time and C₀^’^ represents the contaminant concentration at t = 0 after dark adsorption equilibrium (30 min ultrasound).

## 3. Results

**Figure 2:**
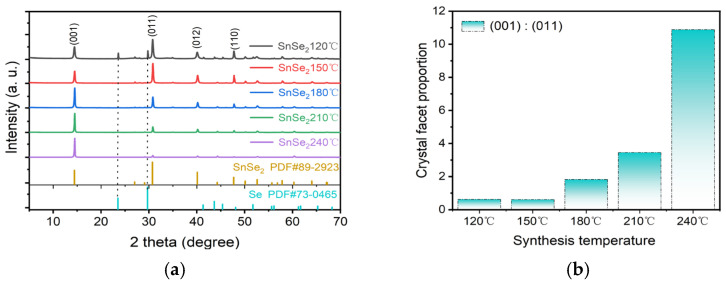
The XRD patterns of (**a**) SnSe_2_ materials synthesized at different temperatures; (**b**) the ratio of the (001) to (011) crystal plane intensities.

The crystal structure of the synthesized photocatalyst was characterized by XRD patterns in Figure 2a. SnSe_2_ samples prepared at various hydrothermal temperatures exhibited distinct diffraction peaks at 14.47°, 30.78°, 40.08°, and 47.73°, corresponding to the (001), (011), (012), and (110) planes of hexagonal SnSe_2_ (PDF#89-2939). The sharpness of the primary peaks confirmed the high crystallinity and purity of all SnSe_2_ samples. However, impurity peaks emerged in the sample synthesized at 120 °C. Comparative analysis with hexagonal Se reference (PDF#73-0465) revealed that the impurity peaks at 23.56° and 29.75° matched the (100) and (101) planes of elemental Se, indicating incomplete reaction at low temperatures. The XRD patterns demonstrated progressive disappearance of impurity peaks with increasing temperature, accompanied by facet-dependent evolution between (001) and (011) planes. We further clarify that the synergistic effect between heating temperature and substrate temperature plays a critical role in regulating phase composition and microstructure during crystal growth. Temperature mediates the structural evolution of SnSe_2_ through mechanisms such as thermal expansion, decomposition reactions, and phase transitions. Among these, high-temperature decomposition and phase transitions act as dominant factors driving phase alterations, while thermal expansion and disordering primarily influence lattice parameters and transport properties. In this study, we highlight a key discovery: high-temperature-induced random interlayer rotation significantly alters the exposure degree of distinct crystal facets in SnSe_2_ [31]. As shown in Figure 2b, the intensity ratio of (001) to (011) peaks remained almost constant 3:5 between 120 °C and 150 °C. Above this range, the (001) peak intensity progressively increased while the (011) intensity decreased, resulting in an increase in the (001)/(011) ratio from 3:5 at 150 °C. These results establish hydrothermal temperature as a critical factor governing the relative exposure of (001) and (011) facets, with higher temperatures favoring dominant (001) facet orientation.

**Figure 3:**
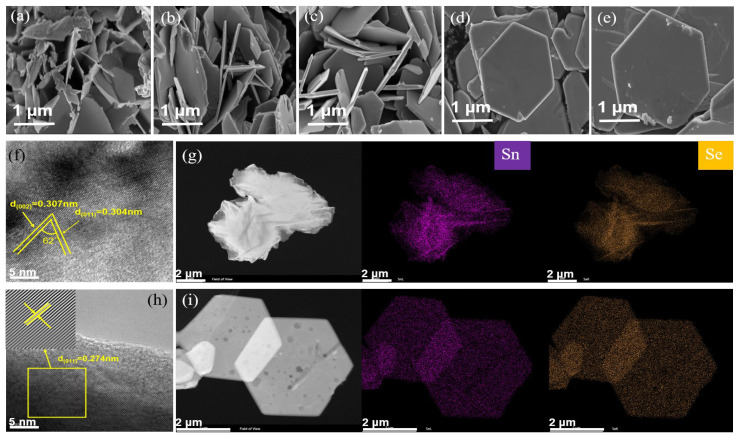
(**a**–**e**) SEM images of SnSe_2_ synthesized at hydrothermal temperatures of 120 °C, 150 °C, 180 °C, 210 °C, and 240 °C, respectively; lattice fringes observed by TEM for SnSe_2_ prepared at 120 °C (**f**) and 240 °C (**h**); EDS elemental mapping images corresponding to the 120 °C (**g**) and 240 °C (**i**) samples illustrate the microstructure of the material.

Figure 3 illustrates the morphology and microstructure evolution of SnSe_2_ synthesized at varying hydrothermal temperatures. Representative SEM images (Figure 3a–e) reveal distinct structural transformations: at 120 °C, SnSe_2_ forms irregular flower-like clusters with undefined edges, while interlocked hexagonal layered structures emerge through dense stacking at 150 °C; above 150 °C, well-defined hexagonal plates (edge length ≈ 2 μm) dominate, exhibiting geometric perfection. This morphological progression aligns with the XRD-determined crystallographic evolution. High-resolution TEM analysis (Figure 3f) of the sample at a hydrothermal temperature of 120 °C shows lattice fringes with spacings of 0.307 nm and 0.304 nm, which are well indexed to the (002) and (011) planes, respectively. The measured interplanar angle of 62° closely matches the theoretical value (61.73°). The sample of 240 °C (Figure 3h) displays fully developed hexagonal plates with interlocking stacking, predominantly exposing (011) facets. In contrast, samples synthesized at 210 °C and 240 °C (Figure 3i) exhibit flat-lying hexagonal plates with highly oriented (001) facets. Under high-temperature conditions, thermal expansion induces an increase in interlayer spacing, leading to a reduction in van der Waals forces and consequently weakening the bonding strength between (001) planes. This results in greater exposure of (001) facets at elevated temperatures [32,33]. The lattice coherence observed in defect-free regions of both low-temperature (Figure 3f) and high-temperature samples (Figure 3h) confirms successful synthesis of phase-pure 2D SnSe_2_. Elemental mapping images (Figure 3g–i) demonstrate the homogeneous distribution of Sn and Se atoms across the hexagonal plates, confirming structural integrity.

**Figure 4:**
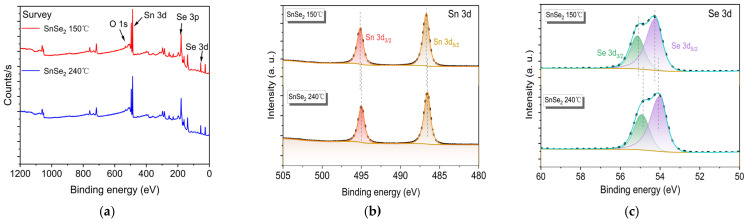
Survey spectrum of SnSe_2_ (**a**); Sn 3d (**b**); Se 3d (**c**).

XPS survey scan (Figure 4a) reveals Sn 3d, Se 3, and Se 3p characteristic peaks, confirming the stable formation of Sn-Se compounds. Identification of Se^2−^ chemical state via Se 3p orbital satellite peak. High-resolution Sn 3d spectra (Figure 4b) for samples synthesized at 150 °C and 240 °C reveal contributions from Sn⁴⁺ states. The dominant peaks at 486.7 eV (Sn 3d_5/2_) and 495.1 eV (Sn 3d_3/2_) with a spin-orbit splitting of 8.4 eV confirm Sn^4+^ as the primary oxidation state. The Se 3d spectrum (Figure 4c) exhibits characteristic doublets at 54.3 eV (3d_5/2_) and 55.0 eV (3d_3/2_) [34], consistent with Se²⁻ anions [35,36]. The positive binding energy shifts observed in XPS spectra originate from reduced electron cloud density around oxidized species. Electron depletion enhances nuclear attraction to core electrons, thereby increasing binding energies [37]. Intriguingly, the Se 3d peaks for the sample of 150 °C show 0.3–0.5 eV upshifts compared to those of the 240 °C counterparts, suggesting enhanced electron withdrawal. This elevated oxidation capacity likely improves surface adsorption and charge carrier separation efficiency. Collective XPS evidence corroborates the successful synthesis of SnSe_2_ and establishes temperature-dependent electronic structure modulation.

**Figure 5:**
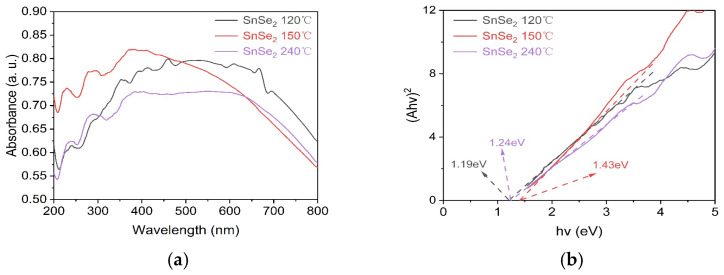
UV–Visible diffuse reflectance spectra (**a**); bandgap (**b**).

The energy band structure of semiconductors critically governs their light-harvesting capacity and photocatalytic efficacy. UV–Vis diffuse reflectance spectra of SnSe_2_ synthesized at 120 °C, 150 °C and 240 °C are shown in Figure 5a. All three samples exhibited broad light absorption ranges with absorption edges extending beyond 800 nm. Notably, the SnSe_2_ prepared at 150 °C demonstrated stronger absorption in the ultraviolet region (200–450 nm) compared to those synthesized at 120 °C and 240 °C. Furthermore, The optical bandgaps of SnSe_2_ synthesized at 120 °C, 150 °C and 240 °C were calculated using the Tauc relation (*αhν*)^1/2^ = *k*(*hν − E_g_*), consistent with its direct bandgap semiconductor nature (*n* = 1/2) [38,39]. As shown in Figure 5b, the derived bandgap values were 1.19 eV (1042 nm), 1.43 eV (867 nm), and 1.24 eV (1000 nm) [40], respectively, with absorption edges located in the near-infrared region. Notably, although the Se impurities formed under 120 °C reaction conditions result in a reduced bandgap for the SnSe_2_ material, the UV-range absorption exhibits weaker intensity compared to samples synthesized at 150 °C reaction conditions [41]. The 150 °C sample exhibited the largest bandgap yet demonstrated stronger ultraviolet absorption (200–450 nm) (see Figure S1, Supplementary Materials). This enhanced absorption likely promotes the generation of high-energy electron-hole pairs, thereby improving redox activity and photocatalytic efficiency.

**Figure 6:**
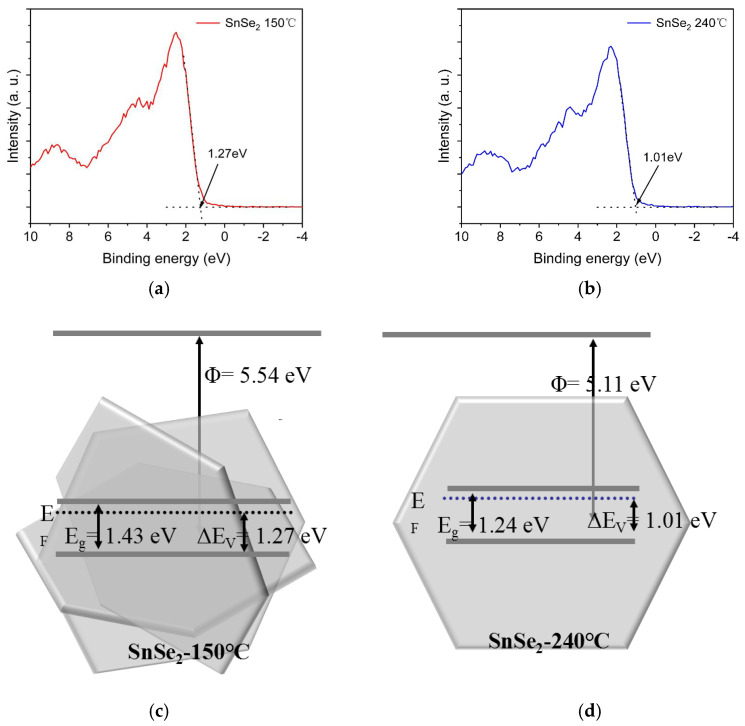
XPS valence band spectra of SnSe_2_ (**a**,**b**); the energy band diagrams of SnSe_2_ synthesized at hydrothermal temperatures of (**c**) 150 °C and (**d**) 240 °C.

Figure 6a,b show the valence band spectra of SnSe_2_ synthesized hydrothermally at 150 °C and 240 °C, respectively. As shown in Figure 6c,d, oxidation reactions play a critical role in the photocatalytic degradation of dye. The analysis reveals that the (011) facet of SnSe_2_ synthesized at 150 °C exhibits a higher valence band maximum compared to the (001) facet formed at 240 °C. This indicates the stronger oxidative capacity of the holes in the (011) facet. During photocatalytic reactions, valence band holes in the (011) facet oxidize H₂O or OH^−^ to generate hydroxyl radicals (OH) and other reactive oxygen species (O₂^−^). These radicals degrade dye molecules into small compounds such as CO₂ and H₂O through direct oxidation or via intermediate reactions, thereby accelerating dye decomposition [42,43].

**Figure 7:**
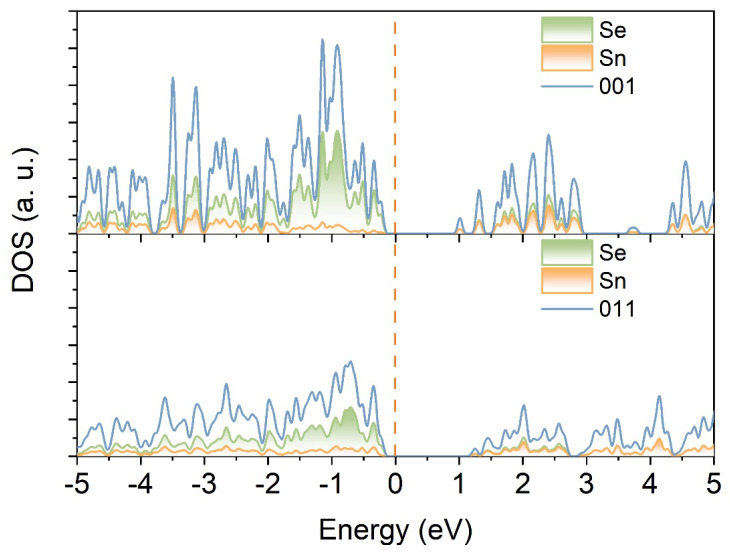
Comparative structural diagrams of the (001) and (011) planes of SnSe_2_.

To further investigate the band structure, density functional theory (DFT) calculations were performed to determine the density of states (DOS) for the (001) and (011) planes of SnSe_2_ (as show as Figure 7). The bandgaps for these planes were 1.22 eV and 1.4 eV, respectively, with corresponding D-band centers at −0.344 eV and 0.128 eV. Analysis of the bandgaps and D-band centers revealed that the (011) plane exhibits a Fermi level closer to the D-band center, indicating a higher degree of d-electron state filling. This enhanced filling likely facilitates the formation of additional active catalytic sites. The (011) facet demonstrates a 0.04 eV enhancement in adsorption energy *Eads = Etotal – ESnSe_2_ – E(MG)* compared to the (001) facet, coupled with a + 0.14 eV charge transfer difference at the interfacial region (see Figure S4, Supplementary Materials) [44]. This interfacial energetic superiority is governed by local electron density redistribution. In the case of MG, the elevated Eads and directional charge transfer pathways act synergistically to accelerate surface activation kinetics.

**Figure 8:**
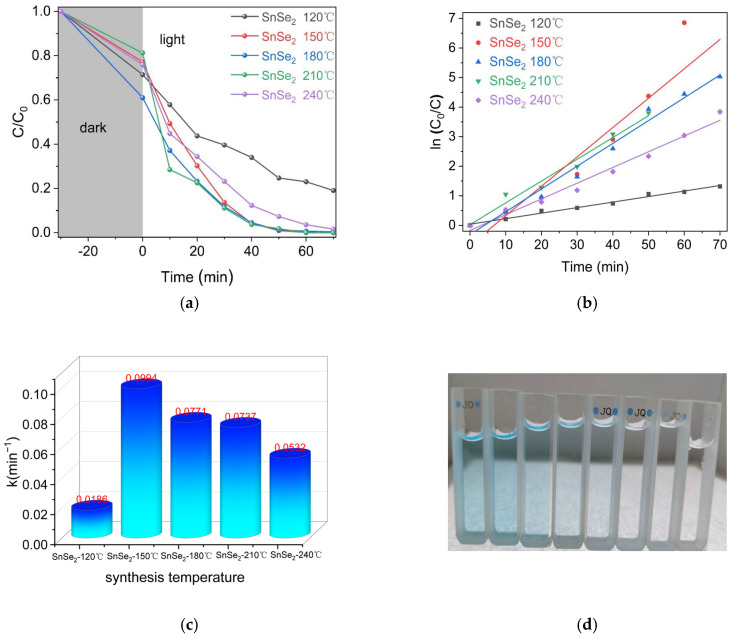
(**a**) Photocatalytic efficiency of SnSe_2_ for MG degradation under varying hydrothermal temperatures; (**b**) corresponding pseudo-first-order kinetic plots; (**c**) comparative visualization of the pseudo-first-order rate constants k; (**d**) time-dependent photocatalytic degradation profile of MG by SnSe_2_ synthesized at 150 °C, sampled at 20 min intervals.

Figure 8 illustrates the photocatalytic activity of SnSe_2_ synthesized at different hydrothermal temperatures.

As shown in Figure 8a, SnSe_2_ synthesized between 150 °C and 210 °C demonstrated superior photocatalytic performance, achieving complete dye degradation (100%) within 60 min. In contrast, samples prepared at 240 °C and 120 °C showed reduced efficiencies of 63% and 77%, respectively, while the 180 °C sample exhibited 99% degradation. The sample at 120 °C exhibited the lowest degradation rate of MG dye is probably attributable to the incorporation of Se impurity into the sample, reducing its photocatalytic capacity.

The fitted rate constants (Figure 8c) were 0.018, 0.099, 0.073, 0.077 and 0.053 min⁻¹ for samples synthesized at 120 °C, 150 °C, 180 °C, 210 °C and 240 °C, respectively. The 150 °C sample demonstrated a rate constant 5.5 times higher than that of the 120 °C sample and 1.9 times greater than the 240 °C sample. This enhanced performance correlates with the predominant exposure of (011) crystal facets (Figure 2b), which facilitate efficient charge separation and surface reactions. Notably, the facet-engineered SnSe_2_ in this work demonstrates superior photocatalytic degradation efficiency compared to previously reported SnSe_2_-based systems (see Table S1, Supplementary Materials).

**Figure 9:**
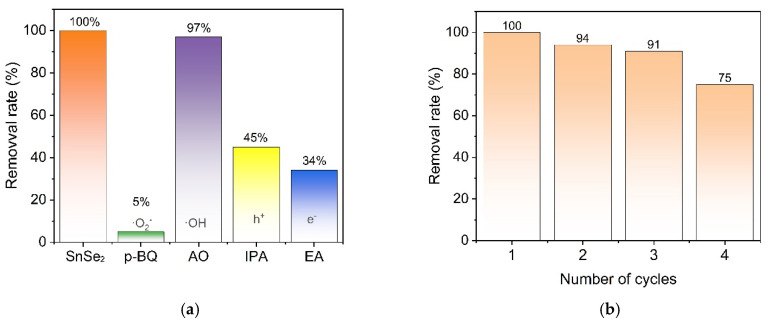
(**a**) Scavenger studies; (**b**) recyclability tests.

As shown in Figure 9a, to identify the dominant active species in the photocatalytic degradation of MG, ethanol (EA), isopropanol (IPA), p-benzoquinone (p-BQ), and ammonium oxalate (AO) were employed as scavengers for electrons (e⁻), holes (h⁺), superoxide radicals (O₂⁻), and hydroxyl radicals (OH), respectively. The effects of these scavengers on photocatalytic performance were systematically evaluated. In the photocatalytic degradation experiment without scavengers, the degradation efficiency of MG reached 100% within 60 min. However, when EA, IPA, p-BQ, and AO were added, the degradation efficiencies decreased to 34%, 45%, 5%, and 97%, respectively. These results indicate a significant generation of ·O₂⁻ radicals, formed via the reaction between electrons (e⁻) and dissolved O₂, which play a dominant role in the catalytic degradation process. When h⁺ and OH scavengers were introduced, it was observed that ·OH radicals were generated from the reaction of h⁺ with H₂O. This suggests that while h⁺ alone can directly contribute to MG degradation, their efficiency is lower compared to ·O₂⁻. Most h⁺ participated directly in the photocatalytic process rather than converting to OH radicals, which explains the weaker inhibition observed with ·OH scavengers [45]. Additionally, four consecutive photocatalytic cycles were conducted (Figure 9b). The catalyst retained 75% of its initial degradation efficiency after four cycles, demonstrating that the SnSe_2_ photocatalyst maintains reasonable stability and reusability despite partial loss during recovery. Simultaneously, by simulating the presence of Cl⁻ and SO₄²⁻ ions in industrial wastewater, we verified whether the prepared photocatalyst was affected when degrading MG. The results demonstrate that the addition of these two ions does not significantly influence the degradation, thereby proving the robustness and practical significance of our photocatalyst (see Figure S3, Supplementary Materials).

**Figure 10:**
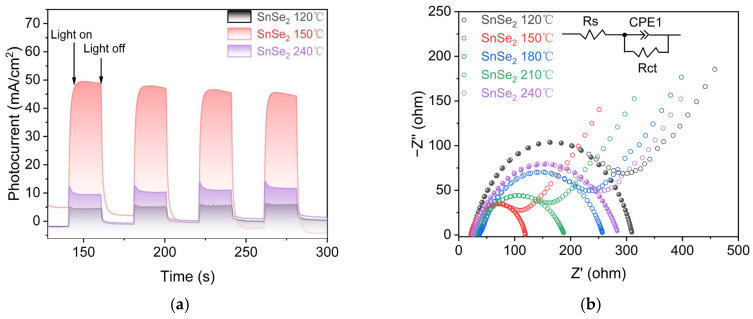
Electrochemical transient photocurrent response (**a**); Nyquist plot (**b**).

Figure 10a shows transient photocurrent responses under light illumination. The 150 °C SnSe_2_ sample exhibited the highest photocurrent density, surpassing the 120 °C and 240 °C samples by factors of 8.9 and 4.3, respectively. Enhanced photocurrent indicates reduced charge recombination and more efficient charge transfer, leading to improved photon utilization and photocatalytic activity. Figure 10b demonstrates charge carrier mobility through electrochemical impedance analysis. The 150 °C SnSe_2_ displayed the smallest arc radius in Nyquist plots, correlating with superior charge separation and transport efficiency. Reduced charge recombination and accelerated electron transfer kinetics explain its enhanced photocatalytic reaction rates. Combined, these results confirm that the 150 °C SnSe_2_ achieves optimal photocatalytic performance due to rapid charge carrier migration and minimized recombination losses, enabling efficient utilization of photoexcited charges.

**Figure 11:**
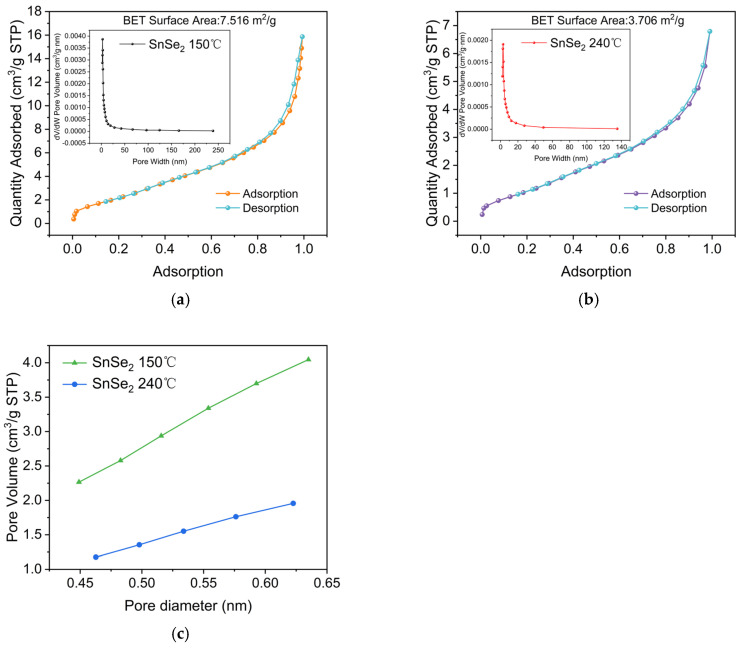
(**a**) N₂ adsorption–desorption isotherms, BET and BJH of SnSe_2_-150 °C; (**b**) N₂ adsorption–desorption isotherms, BET and BJH of SnSe_2_-240 °C; t-plot (**c**).

Figure 11a,b displays Type IV nitrogen adsorption–desorption isotherms with H3-type hysteresis loops for hydrothermally synthesized SnSe_2_ at 150 °C and 240 °C. The BET surface area analysis reveals that the 150 °C sample (7.516 m²/g) exhibits 2.03 greater specific surface area than the 240 °C counterpart (3.706 m²/g). This substantial enhancement provides abundant active sites for photocatalytic degradation reactions. Comparative pore size distribution analysis demonstrates: SnSe_2_-150 °C exhibits unimodal mesoporous dominance (2.18–238.82 nm range) with a primary peak at 2.71 nm (0.00387 cm³ g⁻¹ nm⁻¹ peak volume). The sharp pore volume decline beyond 50 nm confirms mesoporous structure prevalence. SnSe_2_-240 °C shows narrowed distribution (2.01–135.21 nm) with peak shifting to 2.85 nm (0.00191 cm³ g⁻¹ nm⁻¹). Total pore volume decreases by 50.7% compared to the 150 °C sample. These structural changes suggest high-temperature hydrothermal processing induces pore coalescence/collapse. Consequently, the 150 °C-derived material demonstrates superior adsorption capacity for bulky molecules due to its broader pore distribution, whereas the 240 °C counterpart shows compromised photocatalytic performance in adsorption-dependent systems. t-Plot analysis (Figure 11c) confirms a 29.2% external surface area reduction at 240 °C, consistent with BET trends. Importantly, despite its lower surface area, the 240 °C sample achieves enhanced thermal stability through preferential exposure of thermodynamically stable (001) crystal facets. This work establishes a hydrothermal temperature regulation mechanism: low-temperature synthesis (150 °C) optimizes surface properties for adsorption-driven photocatalysis. High-temperature processing (240 °C) enhances structural stability via crystal facet engineering.

## 4. Conclusions

To overcome the limitations of conventional heterojunction-dependent photocatalysts, we synthesized phase-pure SnSe_2_ using a simple hydrothermal method by modulating reaction temperatures. Temperature-dependent morphological evolution was observed, with selective facet exposure driven by tailored van der Waals interactions under different synthesis conditions. XRD peak ratios, SEM imaging, and XPS analysis (Sn 3d and Se 3d peak shifts) confirmed that temperature gradients modulate interlayer van der Waals interactions, directly inducing facet-selective growth. The BET surface area and pore size analyses further demonstrated that SnSe_2_ synthesized at 150 °C possesses enhanced structural characteristics that correlated with its superior photocatalytic performance. SnSe_2_ demonstrated exceptional photocatalytic potential for environmental remediation, achieving complete degradation of 40 mg/L MG within 60 min at 150 °C. The (011)-facet-dominated SnSe_2_ outperformed the (001)-facet-rich sample by 1.9-fold, highlighting its superior efficiency. DFT calculations corroborated the enhanced oxidative capacity of the (011) facet, rationalizing its superior photocatalytic activity and aligning with experimental results. This work advances the mechanistic understanding of structure–activity relationships in 2D SnSe_2_ and establishes a design strategy for noble-metal-free 2D photocatalysts.

## Data Availability

Data are contained within the article.

## Supplementary Materials

The following supporting information can be downloaded at: https://www.mdpi.com/article/10.3390/nano15110850/s1.
