# Supplementary material for: Crystal Facet Engineering of 2D SnSe2 Photocatalysts for Efficient Degradation of Malachite Green Organic Dyes
Source: Nanomaterials (Basel). 2025 Jun 2;15(11):850. doi: 10.3390/nano15110850 (PMC12157776; doi:10.3390/nano15110850)
Supplement: Supplementary file 1 [file nanomaterials-15-00850-s001.zip › nanomaterials-3608940-supplementary.pdf]

nanomaterials

Supplementary Materials

Crystal Facet Engineering of 2D $SnSe_2$ Photocatalysts for Efficient Degradation of Malachite Green Organic Dyes

Liying Wen [1,†], Fangfang Cheng [1,†], Xinyu Zhao [1], Lin Han [1,2], Dongye Zhao [1] and Shifeng Wang [1,*]

[1] Key Laboratory of Plateau Oxygen and Living Environment of Xizang Autonomous Region, College of Science, Xizang University, Lhasa 850000, China; wenliying@stu.utibet.edu.cn (L.W.); chengfangfang@stu.utibet.edu.cn (F.C.); zhaoxinyu@stu.utibet.edu.cn (X.Z.); hanlin@stu.utibet.edu.cn (L.H.); zdy@utibet.edu.cn (D.Z.)

[2] School of Ecology, Xizang University, Lhasa 850000, China

* Correspondence: wsf@utibet.edu.cn

† These authors contributed equally to this work.

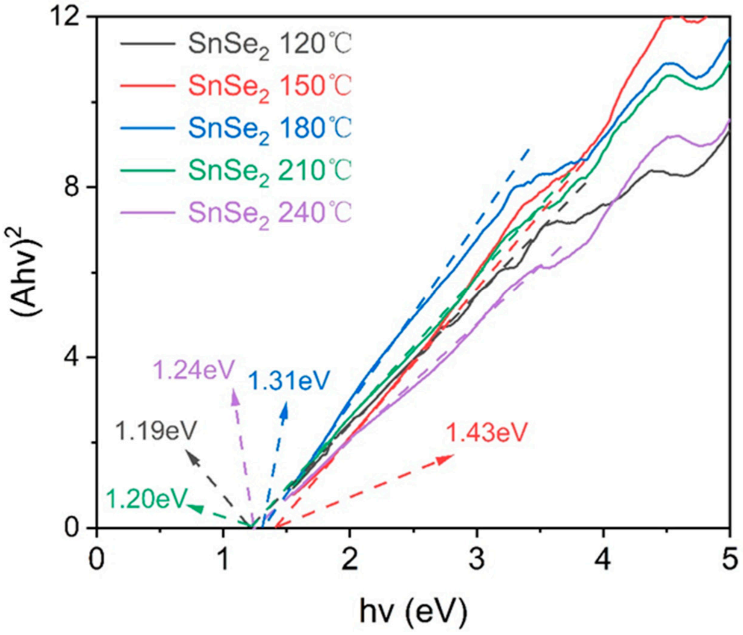

Figure S1. Bandgap.

Academic Editor: Yiannis Deligiannakis

Received: 10 April 2025
Revised: 21 May 2025
Accepted: 31 May 2025
Published: 2 June 2025

Citation: Wen, L.; Cheng, F.; Zhao, X.; Han, L.; Zhao, D.; Wang, S. Crystal Facet Engineering of 2D $SnSe_2$ Photocatalysts for Efficient Degradation of Malachite Green Organic Dyes. *Nanomaterials* **2025**, *15*, 850. https://doi.org/10.3390/nano15110850

Copyright: © 2025 by the authors. Submitted for possible open access publication under the terms and conditions of the Creative Commons Attribution (CC BY) license (https://creativecommons.org/licenses/by/4.0/).

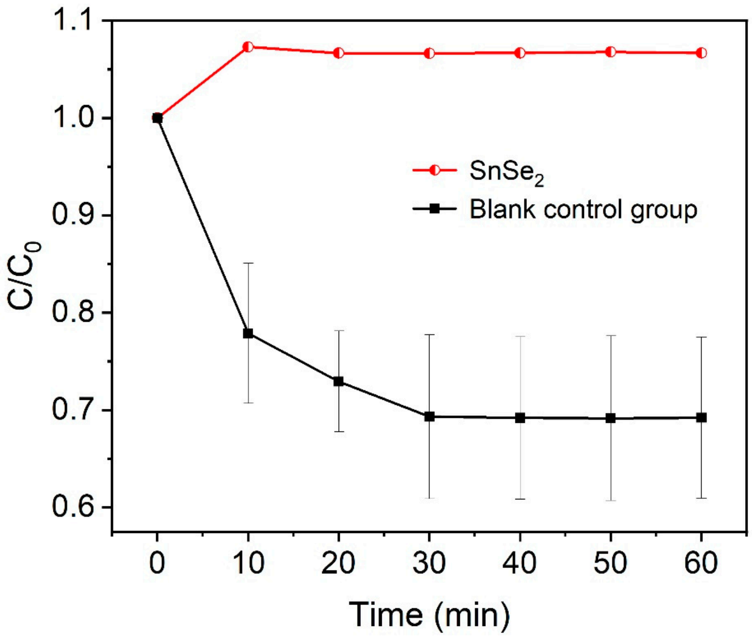

Figure S2. Adsorption-Desorption Equilibrium Profiles Under Dark Conditions.

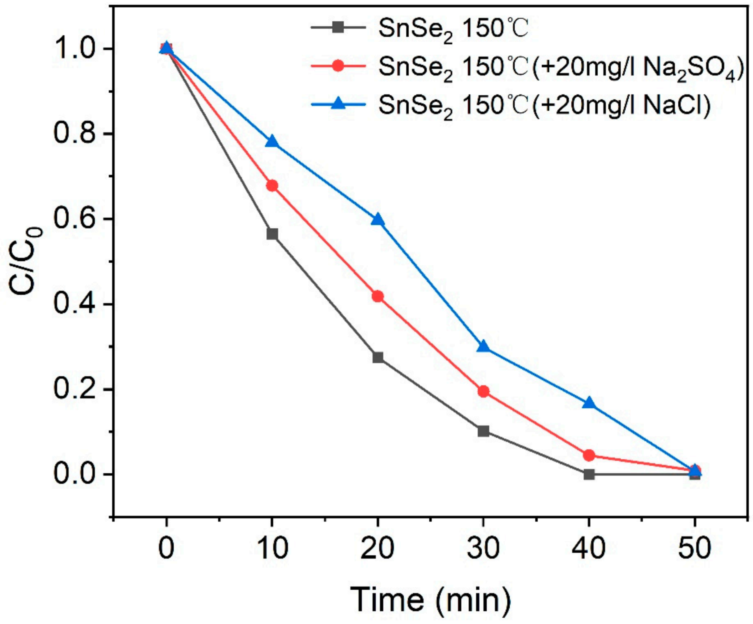

Figure S3. Mechanistic impact of coexisting Cl^-/SO_4^{2-} on MG photocatalytic degradation.

Table S1. Comparison chart of $SnSe_2$ photocatalyst in relevant reports versus the photocatalyst reported in this paper.

	Mode	Dye	Dye concentration	Degradation time	Efficiency	Reference
$SnSe_2$-Se	Photocatalysis	Rh B	5 mg/L	50 min	94%	[1]
$Ag_3PO_4/SnSe_2$	Photocatalysis	Rh B	10 mg/L	50 min	≈100%	[2]
$Se/SnSe_2/TiO_2$	Photoelectrocatalysis	Rh B	10 mg/L	120 min	95.3%	[3]
$SnSe_2$	Photocatalysis	MG	40 mg/L	60 min	100%	This work

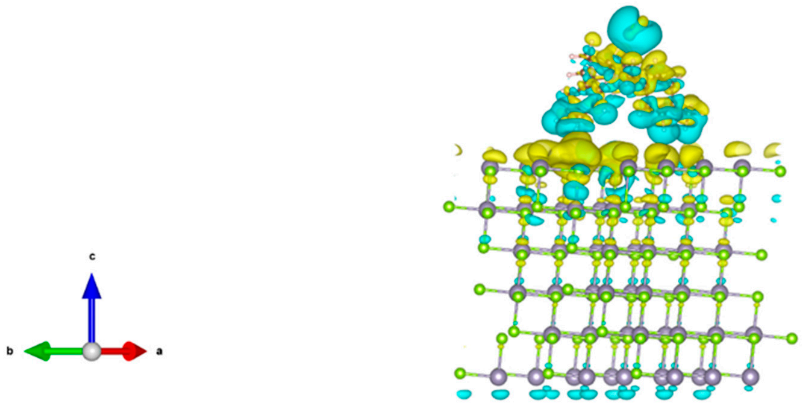

Figure S4. Charge transfer pathway (011).

References

1. Li, J.; Zhao, H.; Lei, Y.; Yang, Q.; Zheng, Z. Synthesis and Photocatalytic Properties of $SnSe_2$ /Se Heterojunction Films. *NANO* **2018**, *13*, 1850045, doi:10.1142/S1793292018500455.
2. Tan, P.; Chen, X.; Wu, L.; Shang, Y.Y.; Liu, W.; Pan, J.; Xiong, X. Hierarchical Flower-like SnSe2 Supported Ag3PO4 Nanoparticles: Towards Visible Light Driven Photocatalyst with Enhanced Performance. *Applied Catalysis B: Environmental* **2017**, *202*, 326–334, doi:10.1016/j.apcatb.2016.09.033.
3. Mu, J.; Luo, D.; Miao, H.; Fan, J.; Hu, X. Synergistic Wide Spectrum Response and Directional Carrier Transportation Characteristics of Se/SnSe2/TiO2 Multiple Heterojunction for Efficient Photoelectrochemical Simultaneous Degradation of Cr (VI) and RhB. *Appl. Surf. Sci.* **2021**, *542*, 148673, doi:10.1016/j.apsusc.2020.148673.
